# Programmable Sequential 4D Actuation in Multistable Kirigami Metamaterials

**DOI:** 10.1002/advs.77412

**Published:** 2026-08-24

**Authors:** Eui‐Hyun Kim, Minsung Kim, Hyunsik Yoon, Keun Park

**Affiliations:** ^1^ Department of Mechanical Design and Robot Engineering Seoul National University of Science and Technology Seoul Republic of Korea; ^2^ Department of Chemical and Biomolecular Engineering Seoul National University of Science and Technology Seoul Republic of Korea

**Keywords:** 4D printing, auxetic metamaterial, bistability, multistable morphing, soft robotics

## Abstract

Programmable shape transformation is essential for adaptive soft mechanisms operating in constrained or dynamic environments. This study presents a modular kirigami metamaterial platform that enables programmable multistability and sequential 4D actuation through thermally triggered recovery. Inspired by geometric motifs from traditional Korean *Dancheong* patterns, in which symmetry and motif stacking govern pattern formation, two kirigami unit cells with different slit configurations are designed to exhibit distinct bistable responses and directional auxetic behavior. Using dual‐material additive manufacturing, each cell integrates thermally responsive and passive thermoplastics to achieve cold‐programmed bistability and sequential 4D actuation. This approach is validated through three functional mechanisms: (i) multimodal bridging structures capable of both mechanically and thermally induced reconfigurations; (ii) multistable 4D grippers capable of inward and outward transformations; and (iii) a biomimetic underwater cleaning robot that autonomously collects and encloses floating debris through sequential thermal recovery. All functions are achieved without electronic or pneumatic actuators, relying entirely on mechanically encoded transformations and thermal activation. Furthermore, the use of thermoplastics provides higher stiffness than elastomer‐based actuators, opening new opportunities for thermo‐mechanically encoded actuation with improved mechanical robustness.

## Graphical abstract

This study presents a modular kirigami metamaterial framework enabling programmable multistability and sequential 4D actuation. By coupling geometry‐controlled multistability with material‐dependent recovery kinetics, spatiotemporal deformation can be precisely programmed. The approach is validated through three functional mechanisms: multimodal transformations, multistable 4D grippers, and a biomimetic underwater cleaning robot, establishing a scalable strategy for electronics‐free actuation in adaptive soft mechanisms.

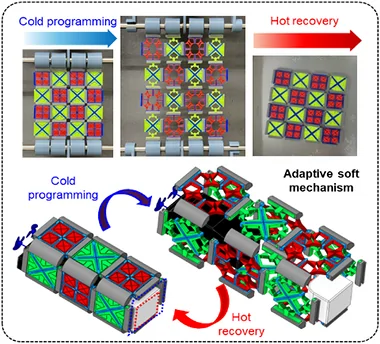

## 1 Introduction

Programmable shape transformation is a fundamental capability for adaptive mechanisms operating in constrained or dynamically changing environments [1]. Conventional adaptive mechanisms typically rely on embedded electronics, pneumatic networks, or external control systems for actuation, which often increase system complexity, power consumption, and operational constraints. In contrast, mechanically encoded structures that autonomously transform their shapes in response to external stimuli offer a promising route toward simple, low‐power, and robust adaptive systems [2].

Four‐dimensional (4D) printing has emerged as a useful strategy for fabricating adaptive structures by integrating stimulus‐responsive materials into additively manufactured architectures [3]. By exploiting smart materials that respond to external stimuli, 4D‐printed structures can undergo programmed shape transformations over time [4]. Among various stimuli‐responsive materials, shape memory polymers (SMPs) have been widely adopted due to their ability to fix temporary shapes and recover their original configurations upon heating above the glass transition temperature (*T_g_*) [5, 6, 7]. Other classes of stimuli‐responsive materials, including liquid crystal elastomers (LCEs) [8] and hydrogels [9], have also been explored for programmable actuation due to their reversible deformation under various stimuli.

Beyond material‐driven actuation, recent studies have increasingly explored the integration of 4D printing with mechanical metamaterials, whose unusual mechanical responses arise primarily from geometrically designed microstructures rather than intrinsic material properties [10]. Among them, auxetic metamaterials exhibiting negative Poisson's ratios have attracted particular attention because of their large deformation capability, enhanced conformability, and tunable mechanical responses [11]. Various auxetic architectures, including re‐entrant lattices and rotating rigid‐unit systems, have been developed to achieve programmable expansion and contraction behaviors [12, 13, 14].

Kirigami structures, derived from the traditional Japanese art of paper cutting, also exhibit auxetic behavior and provide another versatile framework for achieving large, reversible deformations [15]. By introducing patterned cuts into thin sheets, they exhibit high stretchability and complex shape morphing [16]. These characteristics have enabled diverse applications, including soft grippers [17], biomedical devices [18], biomemetic actuators [19], stretchable electronics [20], battery system [21], and compliant robotic mechanisms [22]. When combined with 4D printing, kirigami structures offer a powerful route to programmable actuation by coupling structural mechanics with stimulus‐responsive materials [23].

Another important characteristic of kirigami metamaterials is mechanical bistability, exhibiting two distinct stable configurations separated by an energy barrier [24]. Bistable systems have attracted interest in deployable structures and energy absorption because they can maintain large deformations without continuous energy input [25]. By extending this concept, multistable systems composed of interacting bistable units can exhibit hierarchical deformation pathways and sequential transitions between multiple stable states [26]. Such multistable architectures offer a promising route to encode complex mechanical functions directly into structural geometry [27, 28, 29, 30, 31]. Despite these advances, most kirigami‐based 4D systems primarily focus on single‐mode shape transformations or simple bistable responses. The ability to program sequential, coordinated actuation within modular architectures remains limited, particularly in systems that rely solely on structural design and passive thermal activation without embedded sensors or control electronics [32].

In this study, we present a modular kirigami metamaterial platform that enables programmable multistability and sequential 4D actuation via thermally triggered recovery. Inspired by geometric motifs from traditional Korean *Dancheong* patterns, where symmetry and motif stacking govern pattern formation [33], two kirigami unit cells are designed: a diagonal‐dominant cell (DDC) and an orthogonal‐dominant cell (ODC), which exhibit distinct bistable deformation modes and directional auxetic responses. Using dual‐material additive manufacturing (AM), each unit integrates thermally responsive and passive thermoplastics to achieve cold‐programmed bistability and heat‐triggered recovery. By tailoring kirigami slit geometries, the bistable energy landscape and recovery kinetics can be systematically tuned, enabling programmable actuation sequences within heterogeneous kirigami assemblies. In contrast to conventional SMP‐based 4D printing, which relies on material‐level shape fixing and recovery [5, 6, 7], the proposed kirigami cells perform cold programming through structural bistability rather than material memory, retaining its second stable state without an additional thermal shape‐fixing process.

We demonstrate that composite arrays of DDC and ODC cells exhibit hierarchical deformation modes and controllable multistable transformations through different assembly architectures. Upon heating above the glass‐transition temperature of the active material, these multistable kirigami structures lower their structural confinement and energy barrier, releasing the stored elastic energy to achieve structural 4D recovery. Leveraging these programmable behaviors, we realize mechanically encoded soft mechanisms, including multimodal bridging structures, multistable grippers capable of inward and outward grasping, and an underwater cleaning robot. More importantly, because the recovery sequence is governed by the geometry‐dependent energy landscape rather than by intrinsic material properties alone, the actuation order can be designed a priori through structural programming, providing a predictive framework for spatiotemporal 4D actuation. These results establish modular kirigami metamaterials as a scalable platform for programmable 4D actuation and provide new opportunities for mechanically encoded adaptive systems and untethered soft mechanisms.

## 2 Results and Discussion

### 2.1 Design of Modular Kirigami Cells Inspired by *Dancheong* Motif

**FIGURE 1:**
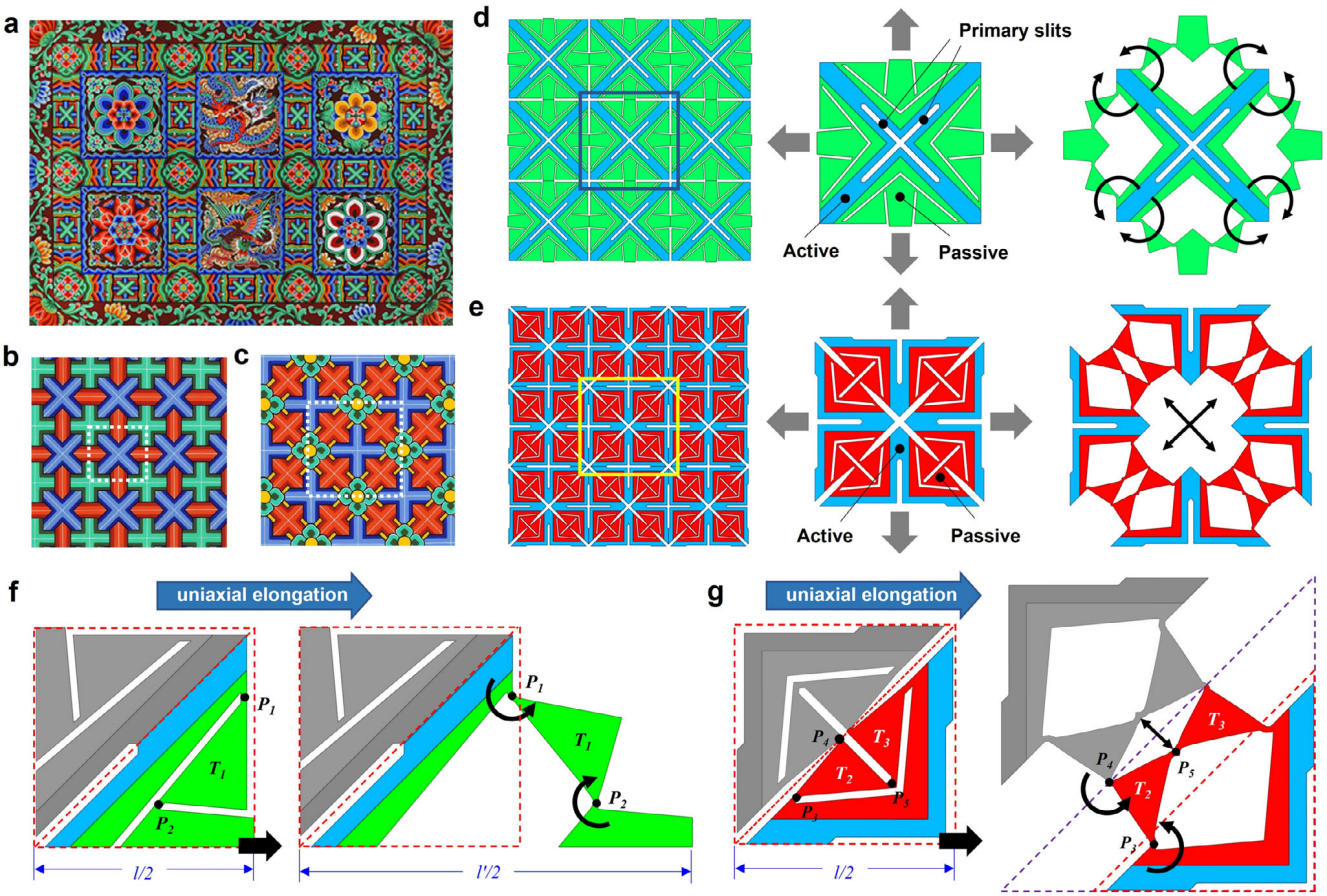
Design and deformation mechanisms of bistable kirigami unit cells inspired by traditional Korean *Dancheong* motifs. (a) Photograph of *Dancheong* patterns on the ceiling of a Korean temple, featuring symmetric and repetitive square‐based motifs. (b) Diagonal‐dominant motif composed of square tiles with intersecting diagonal elements, serving as the geometric inspiration for the diagonal‐dominant cell (DDC). (c) Orthogonal‐dominant motif composed of square tiles and grid‐aligned segments, used to derive the orthogonal‐dominant cell (ODC). (d) DDC design exhibiting bidirectional expansion under biaxial loading, leveraging diagonally connected active slits. (e) ODC design enabling bidirectional expansion under biaxial loading through orthogonally arranged active slits. (f) Uniaxial deformation of the DDC under horizontal loading, resulting in vertical expansion and zero Poisson's ratio behavior. (g) Biaxial deformation of the ODC under horizontal loading, yielding negative Poisson's ratio behavior via diagonal expansion.

Two modular kirigami unit cells are designed by abstracting geometric motifs from *Dancheong*, a traditional Korean decorative painting commonly found in cultural heritage sites. As illustrated in Figure 1a, *Dancheong* patterns employ five symbolic colors (blue, white, red, black, and yellow) that reflect directional and cosmological meanings [33] and are typically arranged as square tilings combined with orthogonal or diagonal line patterns [34]. These patterns can be interpreted as rule‐based geometric systems, where symmetry analogous to paper‐folding and the stacking order of motifs govern pattern formation [35].

From diverse *Dancheong* typologies, two representative patterns were selected for geometric templates: a diagonal‐dominant motif (Figure 1b) and an orthogonal‐dominant motif (Figure 1c). These motifs were translated into two kirigami unit cells: the diagonal‐dominant cell (DDC) and the orthogonal‐dominant cell (ODC), as shown in Figure 1d,e. In each design, the slit network is aligned with the dominant motif direction, producing distinct deformation pathways. Both cells are further partitioned into active and passive material regions, inspired by the color segmentation and rule‐based structure of *Dancheong* patterns [35]. In this configuration, the slit geometry primarily governs cold‐programmed bistability, whereas the active–passive material layout enables thermally triggered recovery.

The DDC incorporates two diagonal active regions forming an X‐shaped configuration (Figure 1d). Two primary slits are embedded within these active domains, while additional arrowhead‐shaped slits are placed in the surrounding passive frame. Under horizontal loading, the structure undergoes anisotropic expansion driven by rotational motion of the inner triangular region (*T_1_*) about hinge points *P_1_* and *P_2_* (Figure 1f). When the applied force exceeds a critical threshold, this motion induces a snap‐through transition to a secondary stable state. Notably, the transformation occurs without vertical deformation, leading to a zero Poisson's ratio (ZPR).

In contrast, the ODC features orthogonally intersecting active regions, forming a grid‐like architecture (Figure 1e). Slits aligned along horizontal and vertical axes are located within the active regions, while additional diagonal cuts span both material domains. During uniaxial stretching, triangular regions *T_2_* and *T_3_* rotate symmetrically about hinge points *P_3_* and *P_4_* (Figure 1g), producing lateral expansion together with axial elongation. This coordinated deformation results in an effective negative Poisson's ratio (NPR).

Consequently, the DDC and ODC designs provide modular kirigami building blocks with distinct bistable deformation modes. Their key design parameters (Figure S1) were tuned through finite element analyses (FEAs) to achieve stable snap‐through transitions with sufficiently high energy barriers and critical forces. These tunable bistable characteristics enable the assembly of multistable kirigami architectures capable of sequential and programmable shape transformations.

### 2.2 Cold‐Programmed Bistability in Auxetic Kirigami Cells

**FIGURE 2:**
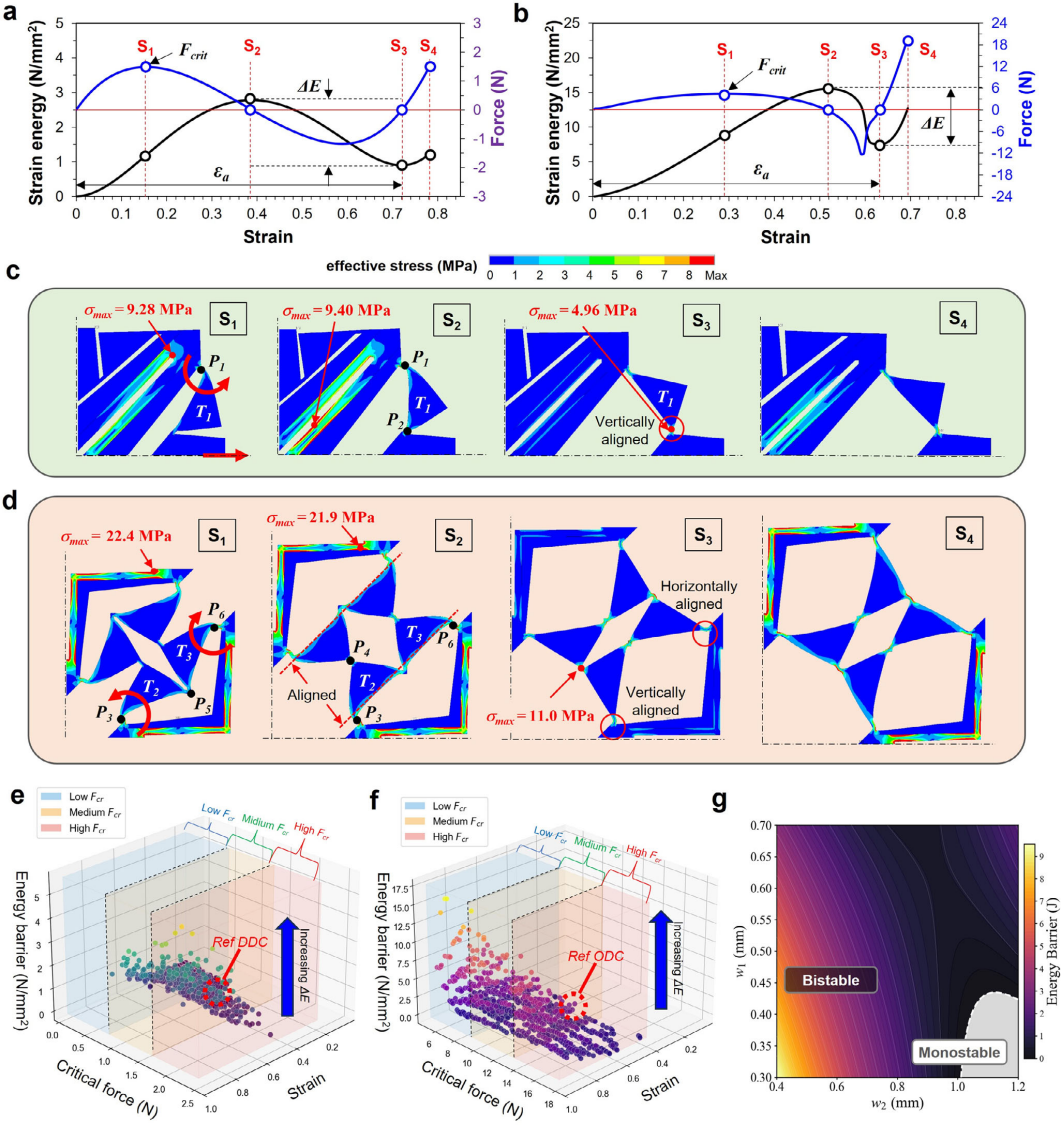
Bistable deformation behaviors of kirigami unit cells under uniaxial extension. (a) Force‐displacement and energy‐displacement curves of the diagonal‐dominant cell (DDC), with key bistable states labeled S_1_–S_4_ (S_1_: maximum force; S_2_: maximum strain energy; S_3_: minimum strain energy; and S_4_: additional 1.0 mm elongation). (b) Corresponding force‐displacement and energy‐displacement curves of the orthogonal‐dominant cell (ODC), indicating higher critical force and narrower bistable range compared to the DDC. (c) Sequential deformation and effective stress distributions of the DDC at four representative states (S_1_–S_4_), highlighting stress concentrations in the active slit and hinge regions during bistable transitions. (d) Sequential deformation and stress distributions of the ODC at states S_1_–S_4_, exhibiting diagonal expansion and distinct hinge alignment mechanisms driving bistability. (e) Responses and design spaces of DDCs with various combinations of geometric parameters (1,138 design cases). (f) Responses and design spaces of ODCs with various combinations of geometric parameters (1,016 design cases). (g) Response surface maps of *ΔE* for ODC (*d_s_* = 0.4 mm, *θ* = 5°, *l_a_* = 10 mm), as a function of *w_1_* and *w_2_* (R^2^ = 0.981).

To quantify cold‐programmed bistability, structural FEA were conducted for the DDC and ODC designs (30 × 30 × 4 mm), under uniaxial tensile loading. Since the present study focuses on in‐plane bistable deformation and recovery, the hinge geometry was designed to minimize out‐of‐plane deformation. Specifically, the hinge width (*w_h_*) was set to 0.4 mm, resulting in a sufficiently high thickness‐to‐width ratio (*t/w_h_* = 10), which provides sufficient resistance to out‐of‐plane displacement and promotes in‐plane hinge rotation as the dominant deformation mode [36]. The resulting force and energy responses are illustrated in Figure 2a,b, with four representative deformation states (S_1_−S_4_) describing the bistable transition and additional elongation. The geometric parameters for DDC and ODC are summarized in Tables S1 and S2, respectively, where the reference parameters of each cell were assigned to FEA models.

For the DDC (Figure 2a), the reaction force reaches a critical value of 1.49 N at S_1_, indicating snap‐through instability. The force subsequently decreases and crosses zero at an apparent strain of 0.38 (S_2_), indicating the onset of instability, and returns to zero again at 0.71 strain (S_3_), corresponding to the secondary stable state. The bistable energy barrier between S_2_ and S_3_ (*ΔE*) is 1.88 N/mm^2^, representing the energy well that stabilizes the second configuration [37].

In contrast, the ODC exhibits a significantly higher resistance to snap‐through (Figure 2b), with a critical force of 4.46 N, approximately three times higher than that of the DDC. The second stable state occurs at a strain of 0.63 (S_3_), and the corresponding *ΔE* reaches 8.02 N/mm^2^, substantially higher than that of DDC. Additionally, the bistable window (S_2_−S_3_) is narrower for the ODC (0.52−0.63) than for the DDC (0.38−0.71), indicating a sharper transition.

The distinct bistable responses originate from different deformation mechanisms, as illustrated by the stress distributions in Figure 2c,d. In the DDC, snap‐through is driven by rotational motion of the inner triangular region (*T_1_*), producing localized stress concentrations near the diagonal active slit during the early deformation stage (S_1_–S_2_). At S_1_, stress concentrates at the tip of the diagonal active slit (*σ_max_* = 9.28 MPa) as the inner triangle *T_1_* rotates counterclockwise around hinge point *P_1_*. This stress concentration arises from the stiffness mismatch between the active and passive regions. As deformation progresses to S_2_, the left edge of *T_1_* aligns vertically, shifting the stress peak toward the active slit body. From S_2_ to S_3_, *T_1_* continues rotating, and the stress is relieved in the slit region while shifting to the lower hinge area. Upon further elongation to S_4_, stresses increase again in both hinges and slit regions, with maximum stresses of 0.83 MPa and 7.20 MPa in the active PLA and passive TPU regions, respectively. These stress levels remain substantially lower than the tensile strengths of PLA (52.5 MPa) and TPU (45 MPa), indicating that the DDC architecture maintains sufficient structural integrity within the investigated deformation range.

In contrast, the ODC exhibits symmetric rotations of triangular regions (*T_2_* and *T_3_*), resulting in distributed stress concentrations along orthogonal hinge locations. At S_1_ and S_2_, peak stresses occur in the orthogonally arranged active slits. As triangles *T_2_* and *T_3_* rotate during uniaxial elongation, their hypotenuses align to form a straight line at S_2_, analogous to the vertically aligned state in the DDC. At S_3_, symmetric rotations relieve slit stresses, while hinges *P_3_* and *P_6_* become aligned along both axes, concentrating stress in these regions. Further deformation to S_4_ increases the stress levels in both slit and hinge zones, resulting in maximum stresses of 18.26 MPa and 12.10 MPa in the PLA and TPU regions, respectively. Although these values are higher than those observed in the DDC, they remain considerably lower than the tensile strengths of the constituent material, indicating that the ODC architecture also maintains structural integrity under the additional deformation considered in this study. The detailed stress evolution at multiple hinge points is provided in Figure S2.

To establish a geometry‐based design rule for bistability, the influence of the key geometric parameters on the energy barrier was systematically analyzed. FEAs were performed over a wide range of geometric parameter combinations, and the resulting datasets were used to evaluate three mechanical responses—apparent strain (*ε_a_*), critical force (*F_crit_*), and energy barrier (*ΔE*)—for the DDC and ODC cells, as illustrated in Figure 2e,f, respectively. The design space was categorized into three *F_crit_* regimes (low, medium, and high), and the resulting FEA datasets were organized into geometry–response design maps. The reference DDC and ODC designs were selected within comparable apparent‐strain ranges and intermediate *F_crit_* regimes while retaining sufficiently high *ΔE* to stabilize the secondary configuration.

The FEA‐derived design maps were then used to identify geometric parameters that provide the desired bistable response. For example, the design map for the ODC (Figure 2f) was visualized as a response surface, where *ΔE* is expressed as a function of the two slit widths (*w_1_* and *w_2_*), as shown in Figure 2g. Notably, regions with negative *ΔE* (gray zones) correspond to monostable configurations, where bistability is not achieved. These results demonstrate that bistability emerges only within a bounded geometric design space defined by the slit geometry [38]. This trend is consistent with the stability transition governed by the balance between rotational compliance and confinement stiffness, as described by Yang et al. [39]. using an extended von Mises truss model. Within this design space, increasing the slit width generally increases the energy barrier, thereby stabilizing the secondary stable states, whereas insufficient slit opening leads to monostable behavior. The resulting design maps therefore provide a practical guideline for selecting slit geometries to achieve target bistable characteristics.

The corresponding force‐strain curves for both the bistable and monostable cases are further demonstrated in Figure S3, where monostable designs distinctly exhibit strictly positive reaction force. Based on these observations, tunable bistability can be achieved by selecting geometric parameters within the bistable domain, providing a quantitative design guideline for constructing modular kirigami assemblies with programmable multistable behavior.

### 2.3 4D Recovery of Bistable Kirigami Cells via Thermal Activation

**FIGURE 3:**
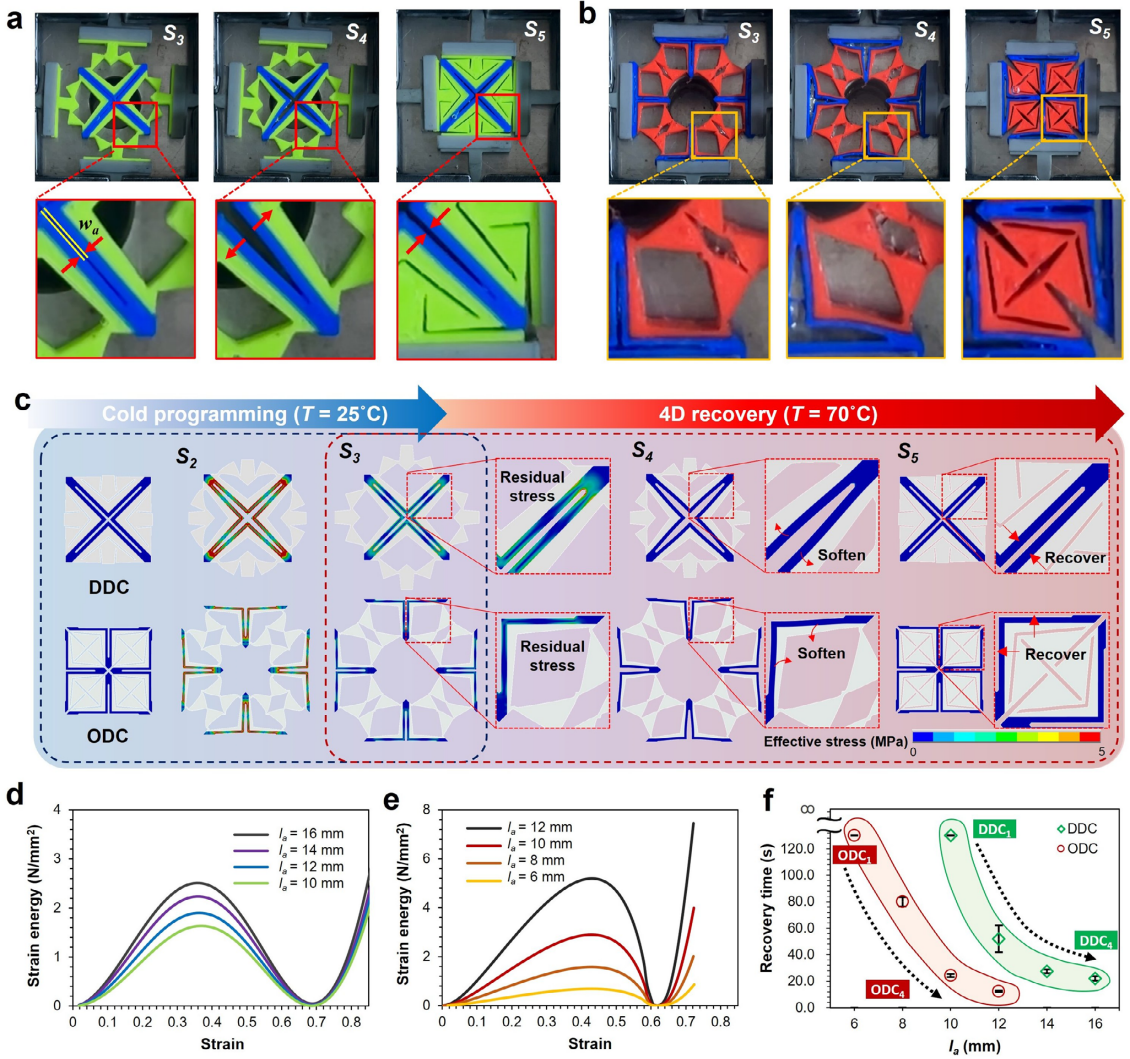
Thermally triggered 4D recovery of cold programmed bistable kirigami unit cells. (a) 4D recovery sequence of a cold‐programmed DDC (*l_a_* = 14 mm) during heating above *T_g_*. (b) 4D recovery sequence of a cold‐programmed ODC (*l_a_* = 10 mm), showing bidirectional recovery behavior. (c) Thermomechanical FEA results illustrating effective stress distributions in the active regions during cold programming and subsequent 4D recovery (states S_2_–S_5_), highlighting stress release and slit widening prior to shape restoration. (d) Effect of active slit length (*l_a_*) on the strain energy within the active‐slit region (DDC). (e) Effect of *l_a_* on the strain energy within the active‐slit region (ODC). (f) Measured 4D recovery times of DDC and ODC unit cells as a function of increasing *l_a_*, demonstrating programmable recovery kinetics.

The thermally triggered recovery of a cold‐programmed DDC_3_ with an active slit length (*l_a_*) of 14 mm is shown in Figure 3a. The bistably expanded cell exhibited negligible out‐of‐plane deformation, with the maximum out‐of‐plane displacement remaining below 3% of the cell thickness throughout the programming and recovery processes. When immersed in a 70°C water bath, the bistably expanded cell returned to its initial configuration within 26 s, indicating that the shape recovery occurred predominantly through in‐plane hinge rotation and slit closure. During heating, the active slit temporarily widened, with the slit width (*w_a_*) increased from 0.6 to 4.72 mm at the intermediate state (S_4_, 23 s), before contracting to 1.34 mm at the recovered state (S_5_). This transient widening of the slit marks the onset of thermally activated recovery.

A similar recovery sequence was observed for the ODC_3_ with *l_a_* = 10 mm (Figure 3b) In this case, *w_a_* increased from 0.6 mm to 1.92 mm at S_4_ (20 s) and subsequently decreased to 0.94 mm at S_5_ (25 s). In both designs, thermal recovery initiates in the active slit regions, where the material softens above *T_g_* (64.4°C). The softened active domains temporarily expand during heating, after which the surrounding passive regions drive inward motion that restores the primary stable configuration.

To elucidate the recovery mechanism, thermomechanical FEAs were conducted (Figure 3c). The simulation captures both the cold‐programming and thermal‐recovery stages, confirming that residual stress remains stored in the bistable configurations after snap‐through (S_2_ and S_3_). When the active regions soften above *T_g_*, stress relaxation drives slit widening and reduces the stored strain energy, enabling the passive domains to retract and complete structural recovery.

Additional FEAs were conducted with *l_a_* varied from 10−16 mm for DDCs and 6−12 mm for ODCs. The corresponding designs are denoted as DDC_1_−DDC_4_ and ODC_1_−ODC_4_, with detailed design parameters and thermomechanical responses summarized in Tables S3 and S4, respectively. As shown in Figure 3d, *ΔE* of the DDC within the active‐slit region increases from 1.63 to 2.46 N/mm^2^ with increasing *l_a_*. A similar trend is observed for the ODC (Figure 3e), where *ΔE* within the active‐slit region rises from 0.69 to 5.19 N/mm^2^. These results indicate that increasing slit length enhances strain energy storage in the bistable configuration, thereby modifying the energetic landscape governing the snap‐through and recovery processes.

Notably, the ODC exhibits a greater sensitivity of *ΔE* to *l_a_* than the DDC. That is, changes in slit length produce a much larger variation in the recovery characteristics of the ODC, whereas the recovery behavior of the DDC changes more gradually. This difference in sensitivity enables the relative recovery rates of the two architectures to be tuned and leads to inversion of the recovery sequence under appropriate geometric conditions. Therefore, the programmable sequential recovery originates from the distinct deformation pathways and strain‐energy evolution of the DDC and ODC architectures rather than from an inherent recovery preference of either architecture.

The influence of geometry on recovery kinetics is shown in Figure 3f, revealing that cells with small slit lengths often failed to recover; for example, DDC_1_ (*l_a_* = 10 mm) and ODC_1_ (*l_a_* = 6 mm) showed no recovery even after 120 s. In contrast, larger *l_a_* values enabled significantly faster recovery, demonstrating that recovery speed can be tuned through geometric design. Importantly, the different recovery rates of DDC and ODC enable programmable actuation sequencing. For instance, pairing DDC_3_ (*l_a_* = 14 mm) with ODC_3_ (*l_a_* = 10 mm) yields comparable recovery times (*t_s_*: 27.4 s for DDC_3_ and 24.3 s for ODC_3_), allowing the ODC to recover slightly earlier. This timing asymmetry provides a key handle for orchestrating sequential multistable transformations in designing composite kirigami assemblies.

The reliability of the bistable transition and recovery performance under repeated thermomechanical loading is presented in Table S5, where the recovered length (*l*_r_), residual strain (ε_r_), and recovery time (*t*_s_) are compared for DDC_3_ and ODC_2_ unit cells. Overall, DDC_3_ exhibited stable performance throughout the five programming–recovery cycles. The average recovery time decreased slightly by approximately 8.5%, while the residual strain remained below 2.65% in all cycles. These results demonstrate robust short‐term bistable‐state retention and stable thermal‐recovery behavior under repeated thermomechanical loading.

In contrast, ODC_2_ exhibited a more pronounced cycle‐dependent response. The average recovery time decreased by approximately 47.8%, whereas the residual strain increased from 6.33% to 8.43%. As the number of cycles increased, the residual strain gradually approached a plateau while the recovery time continuously decreased. These observations indicate a cycle‐dependent evolution of the recovery behavior, characterized by faster recovery together with a moderate increase in residual strain. This trend is associated with a reduction in the effective resistance to the reverse transition after repeated thermomechanical actuation, thereby facilitating the thermally activated recovery process in subsequent cycles.

Notably, all specimens retained the second stable state after removal of the external load and successfully completed thermal recovery in every cycle. Furthermore, no visible fracture of the PLA components or delamination at the PLA–TPU interface was observed after repeated actuation. Although the present experiments were limited to five programming–recovery cycles, the results demonstrate the short‐term reliability of both architectures under repeated thermomechanical loading. Further long‐term cyclic testing will be required to assess fatigue‐induced degradation, bistability retention, and recovery performance over extended service lifetimes.

### 2.4 Programmable Multistability in Modular Composite Kirigami Assemblies

**FIGURE 4:**
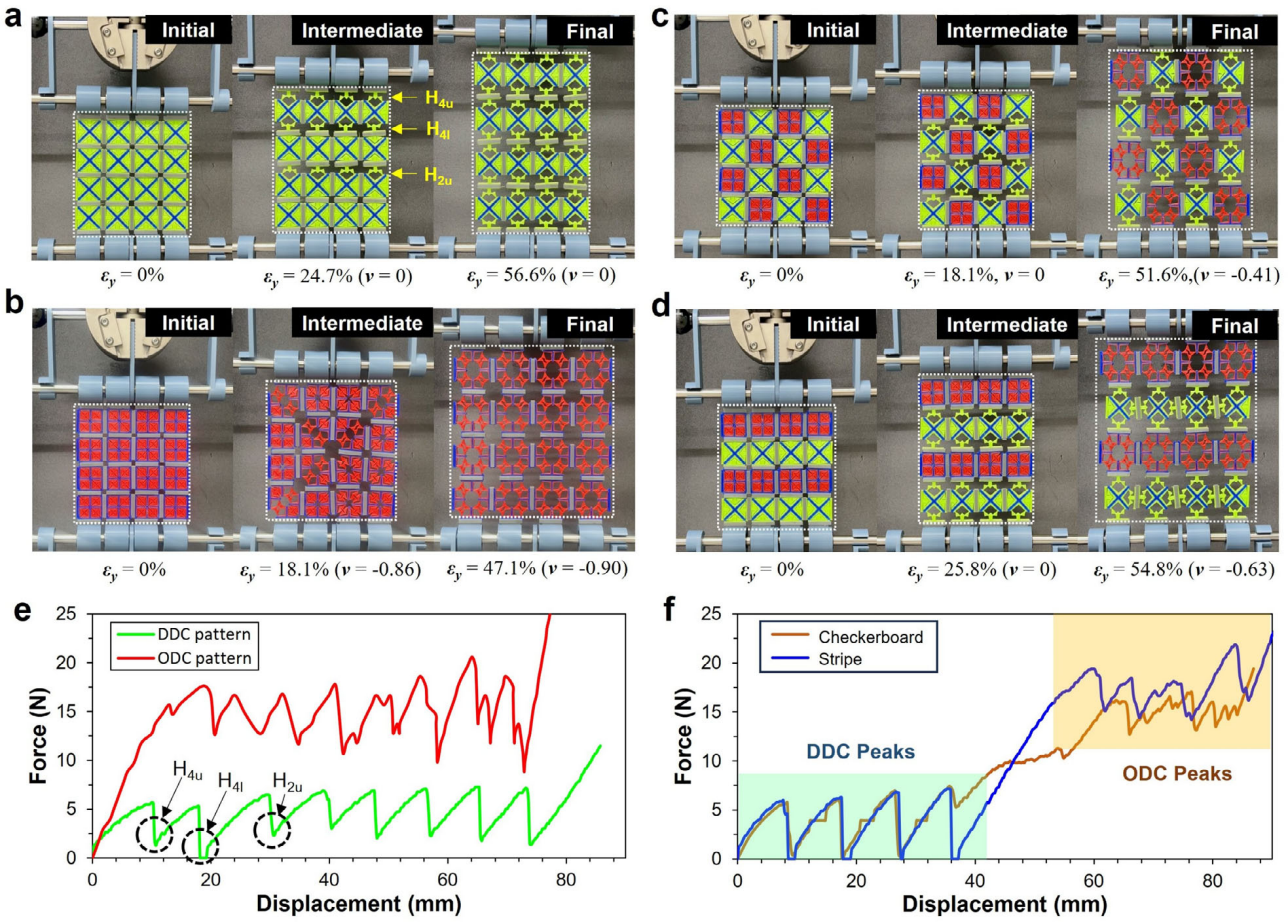
Multistable deformation behaviors of kirigami‐based auxetic arrays with homogeneous and heterogeneous configurations. (a) Sequential bistable deformation of a homogeneous DDC array under uniaxial tension, exhibiting zero Poisson's ratio (ZPR) behavior. (b) Sequential bistable deformation of a homogeneous ODC array under uniaxial tension, showing diagonal expansion and auxetic response with a negative Poisson's ratio. (c) Deformation of a heterogeneous checkerboard structure composed of alternating DDC and ODC cells, demonstrating intermediate ZPR expansion followed by full auxetic transformation. (d) Deformation of a heterogeneous stripe structure with layered DDC and ODC arrays, enabling hierarchical shape transformation from ZPR to enhance auxetic expansion. (e) Force‐displacement responses of homogeneous DDC and ODC arrays, indicating distinct snap‐through characteristics and critical force levels. (f) Force‐displacement curves of heterogeneous checkerboard and stripe composites, highlighting programmable multistability and interaction effects between cell types.

The modular kirigami cells were assembled into homogeneous and heterogeneous arrays to investigate their collective multistable behavior under uniaxial tensile loading. The deformation sequence of a homogeneous DDC_4_ array is presented in Figure 4a and Movie S1. As the applied strain increases, snap‐through transitions occur sequentially across rows of hinges, producing progressive vertical expansion in the order H_4u_, H_4l_, and H_2u_. At the final strain state (*ε_y_* = 56.6%), all primary hinges have transitioned while the lateral width remains nearly constant, resulting in an effective zero Poisson's ratio (ZPR) deformation range.

In contrast, the homogeneous ODC_3_ array exhibited biaxial deformation driven by diagonal snap‐through transitions (Figure 4b and Movie S2). Specifically, the bidirectional bistable transformation initiates along the diagonal direction and progressively propagates throughout the structure. During stretching, coordinated rotations of the kirigami units generate lateral expansion together with axial elongation, producing pronounced auxetic behavior with Poisson's ratios of ‐0.86 at intermediate strain and ‐0.90 at the final deformation state.

The force‐displacement responses of both homogeneous arrays are compared in Figure 4e. The DDC_4_ array displays eight primary force peaks (5.3−7.3 N), each corresponding to sequential snap‐through transitions across the hinge rows. In contrast, the ODC_3_ array exhibits eight major force peaks with additional minor undulations (16.7−20.6 N), reflecting the more complex diagonal deformation pathway and the higher snap‐through threshold of the ODC cell.

It is noteworthy that the local minima in both force–displacement curves remain above zero at the snap‐through positions. Although the reaction force of an individual unit cell approaches zero at its bistable transition point, snap‐through events within the assembled arrays occur sequentially rather than simultaneously. For the DDC_4_ array, the first three local minima appear in the order H_4u_, H_4l_, and H_2u_, indicating that bistable transitions propagate among hinge groups with a time lag. Consequently, while one hinge group undergoes snap‐through, the remaining cells and connecting hinges continue to sustain tensile loading, preventing the overall reaction force from dropping to zero. This effect is more pronounced in the ODC_3_ array because its diagonal and bidirectional transformation pathway results in a larger portion of the structure remaining load‐bearing during each local snap‐through event, thereby generating a higher residual force. Furthermore, friction between the movable holders and guide rods may also contribute to the residual force observed in the experiments by providing additional resistance during the sliding motion required for horizontal deformation.

To explore programmable multistability, heterogeneous arrays were constructed in checkerboard and stripe configurations (Figure 4c,d). In the checkerboard array, the lower‐threshold DDCs activate first, inducing vertical ZPR bistable expansion (Figure 4c and Movie S3). As loading continues, the ODCs undergo snap‐through transitions, triggering diagonal expansion while the DDCs remain in their expanded configurations. This process produces a two‐stage multistable response, in which the structure evolves from a ZPR state to an auxetic NPR state (*ε_y_* = 51.6%, *ν* = ‐0.41).

A similar sequential transformation occurs in the stripe configuration, but with stronger mechanical coupling between layers (Figure 4d and Movie S4). Initially, the DDC layers expand vertically, forming a ZPR state at *ε_y_* = 25.8%. Subsequent activation of the ODC layers induces diagonal expansion that also drives lateral deformation of neighboring DDC layers. This coordinated interaction leads to full auxeticity (NPR), reaching *ν* = ‐0.63 at *ε_y_* = 54.6%, indicating enhanced hierarchical multistability compared with the checkerboard arrangement.

The collective snap‐through behavior of the heterogeneous arrays is reflected in the force‐displacement curves (Figure 4f). In the stripe configuration, the first four peaks (5.8−7.4 N) correspond to the activation of DDC layers, followed by four higher peaks (16.0−21.9 N) attributed to ODC transitions. This clear separation in peak magnitude indicates two distinct transition regimes, governed by different snap‐through thresholds for the constituent cell types. The checkerboard array shows a similar low‐then‐high peak trend, although the transition sequence is more distributed due to stronger geometric coupling between neighboring cells. These results demonstrate that array topology strongly influences the ordering and interaction of bistable transitions, enabling programmable multistable deformation in modular kirigami assemblies.

### 2.5 Programmable Sequential 4D Recovery in Composite Kirigami Assemblies

To demonstrate programmable temporal control over shape recovery, multistably expanded kirigami assemblies were thermally activated to trigger 4D recovery. The recovery sequence was tuned by varying *l_a_*, which controls the recovery kinetics. As shown in Figure 3f, structures with small *l_a_* (DDC_1_ and ODC_1_) showed no observable recovery, whereas larger values resulted in progressively faster recovery. All recovery experiments were conducted by immersing cold‐programmed structures in a 70°C hot‐water bath.

**FIGURE 5:**
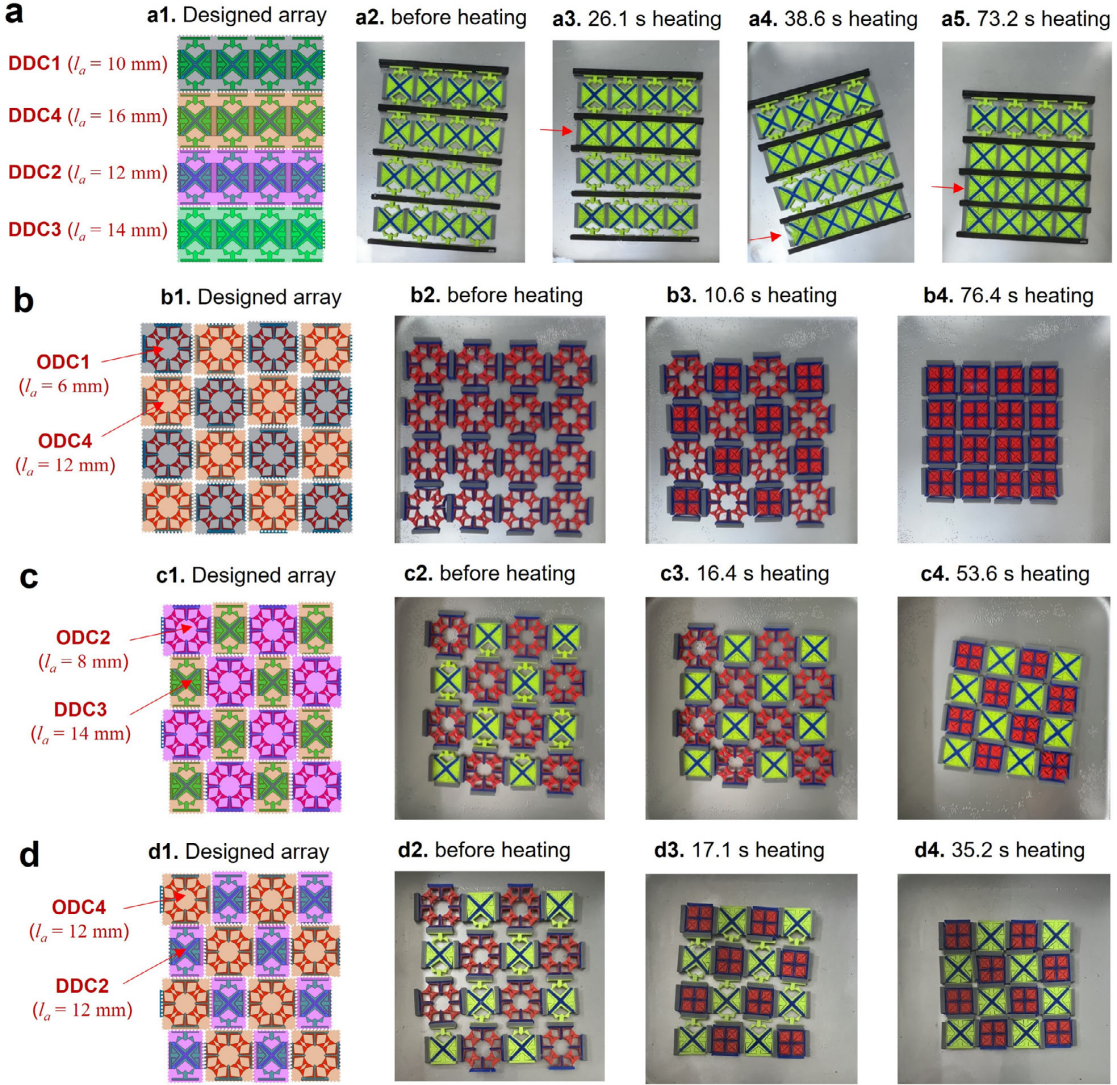
Multistable 4D recovery behaviors of composite auxetic kirigami structures with programmable active slit length (*l_a_*). (a) Uniaxially expanded DDC array with four rows (DDC_1_–DDC_4_), each assigned different *l_a_* (10–16 mm), enabling sequential recovery based on recovery rate. (b) Biaxially expanded ODC array with a checkerboard pattern of two *l_a_* values (ODC_1_: 6 mm, ODC_4_: 12 mm), exhibiting reverse recovery order due to differential thermal response. (c) Checkerboard composite array of DDC_3_ (*l_a_* = 14 mm) and ODC_2_ (*l_a_* = 8 mm), demonstrating a recovery sequence initiating from DDC and followed by ODC. (d) Checkerboard composite array of DDC_2_ and ODC_4_, both with *l_a_* = 12 mm, exhibiting reversed recovery sequence from ODC to DDC. All structures were initially cold‐programmed into their bistably expanded states and thermally activated in a 70°C water bath, showcasing programmable and bidirectional 4D recovery pathways.

The programmable recovery of a 4 × 4 DDC array is shown in Figure 5a, in which each row was assigned a different *l_a_* value: DDC_1_ (*t_s_* > 120 s), DDC_2_ (*t_s_* = 52.0 s), DDC_3_ (*t_s_* = 27.4 s), and DDC_4_ (*t_s_* = 22.0 s). After cold programming into a ZPR bistable configuration (a2), the array recovered sequentially upon heating: DDC_4_ recovered first (a3), followed by DDC_3_ (a4) and DDC_2_ (a5), in descending order of *l_a_*. Notably, the DDC_1_ did not recover even after 73.2 s heating, demonstrating that the recovery sequence can be programmed through the spatial distribution of *l_a_*, with longer slits exhibiting faster recovery kinetics.

A different behavior was observed in the ODC array (Figure 5b). In a checkerboard configuration containing ODC_1_ (*t_s_* > 120 s) and ODC_4_ (*t_s_* = 12.3 s), the cold‐programmed auxetic structure (b2) recovered in the sequence: ODC_4_ recovered first (b3), followed by ODC_1_ (b4). The recovery times observed in the array (10.6 s for ODC_4_ and 76.4 s for ODC_1_) were shorter than the corresponding *t_s_* values measured for the individual unit cells because the array was constrained by the boundary fixtures, as shown in Figure 3b. Nevertheless, the relative recovery kinetics remained unchanged, with cells having longer active slits recovering more rapidly, consistent with the trend observed for the DDC array.

Heterogeneous assemblies combining both cells further demonstrate programmable spatiotemporal actuation (Figure 5c,d). In the checkerboard array shown in Figure 5c, alternating DDC_3_ (*t_s_* = 27.4 s) and ODC_2_ (*t_s_* = 80.0 s) cells produced a DDC‐first recovery sequence upon heating (Movie S5). In contrast, a different checkerboard array was designed to reverse the recovery order of the DDC and ODC, by assigning DDC_2_ (*t_s_* = 52.0 s) and ODC_4_ (*t_s_* = 12.3 s), as shown in Figure 5d. This reversed recovery sequence can also be observed in Movie S6. This reversal originates from the crossover of the recovery‐rate curves for the two cell types (Figure 3f). Although ODC cells recover more slowly at shorter slit lengths, they can recover faster than DDC cells at intermediate slit lengths.

The programmable recovery behavior can be understood from the distinct deformation pathways of the DDC and ODC architectures. The DDC undergoes a relatively simple uniaxial snap‐through process dominated by the sequential rotation of hinges around the primary slit. In contrast, the ODC requires coordinated diagonal and bidirectional compliant rotations of multiple internal triangular elements, resulting in a more complex deformation pathway. Consequently, the strain‐energy landscape of the ODC is considerably more sensitive to variations in *l_a_* than that of the DDC, as evidenced by the larger change in *ΔE* shown in Figure 3e. This difference in sensitivity leads to different recovery kinetics for the two architectures and causes the recovery‐rate curves to intersect (Figure 3f). Therefore, the relative recovery order is not inherently determined by the cell type itself but can be reversed by appropriately selecting the slit lengths of the constituent cells.

Consequently, these results demonstrate that the recovery sequence of composite kirigami assemblies can be bidirectionally programmed by tuning the slit geometry of individual cells. While cold programming follows a mechanically determined sequence governed by the snap‐through thresholds (typically from DDCs to ODCs), thermal recovery can be independently controlled through recovery kinetics. This decoupling of mechanical bistability and thermally triggered recovery enables precise control of both spatial and temporal actuation pathways in modular kirigami metamaterials.

### 2.6 Multimodal Kirigami Mechanisms Enabled by Material–stability Coupling

**FIGURE 6:**
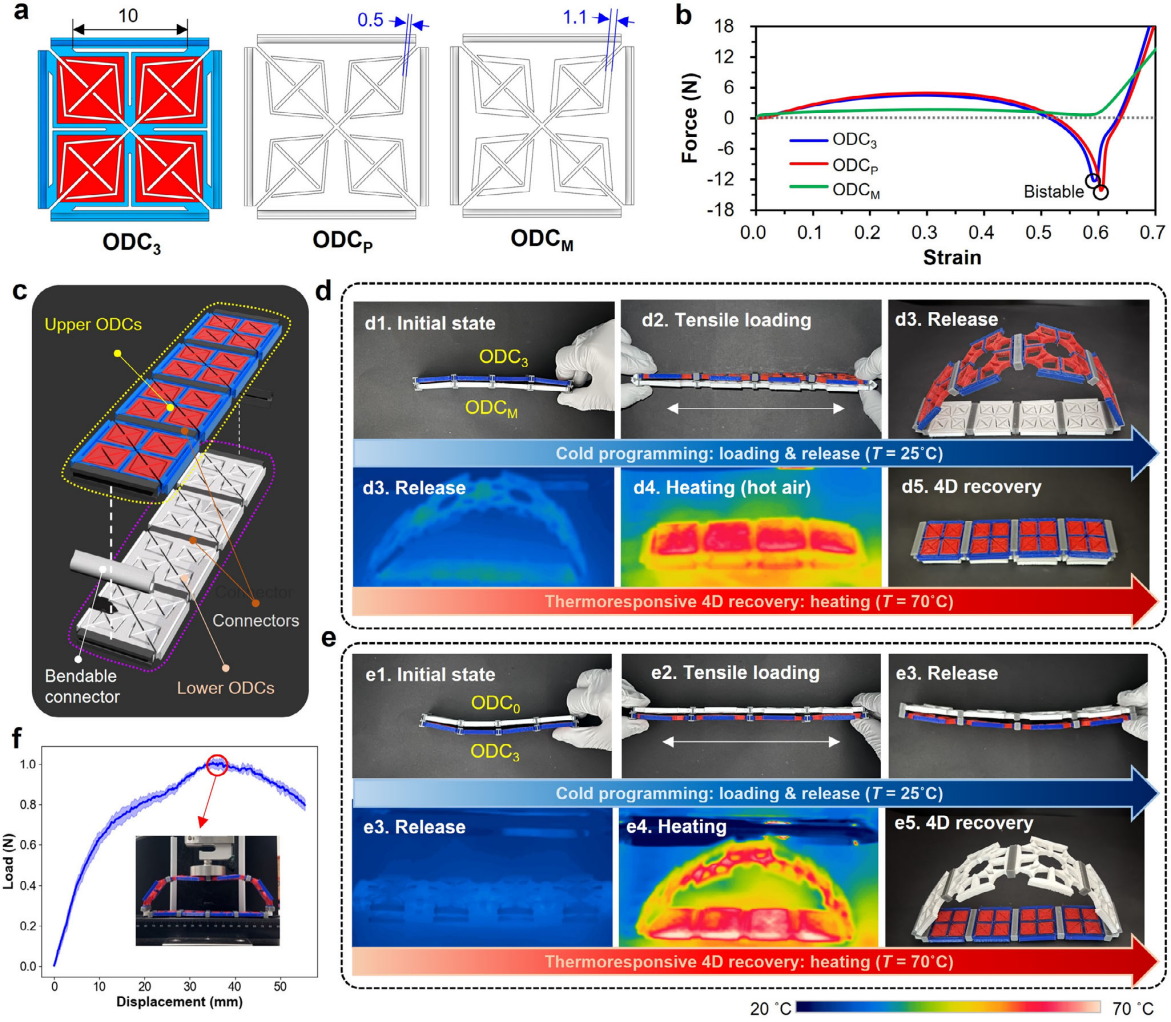
Multimodal kirigami mechanisms enabled by material–stability coupling. (a) Design modification of ODC_3_ into passive only (ODC_P_) and monostable (ODC_M_) configurations (unit: mm). (b) Force‐strain responses of the ODC variants, showing monostable behavior of ODC_M_. (c) Schematic of a double‐layer kirigami bridging structure composed of serially connected ODC units. (d) Cold‐programmed bridging mechanism arising from a mismatch between a bistable dual‐material layer (ODC_3_) and a monostable layer (ODC_M_). (e) Thermally programmed inverse‐bridging mechanism achieved by selective recovery of thermally responsive ODC_3_ and inactive (ODC_P_) layers. (f) Load‐displacement response under compression, showing peak force and negative‐stiffness behavior associated with structural reconfiguration.

Modular bistable kirigami cells were further integrated with monostable counterparts to construct hybrid multilayer mechanisms capable of programmable deformation. To introduce monostable behavior, the dual‐material ODC_3_ was modified into a fully passive bistable cell without an active slit (ODC_P_), as illustrated in Figure 6a. The ODC_P_ cell remains bistable, exhibiting a slightly increased *ΔE* (8.82 N/mm^2^) and *F_crit_* (4.95 N), as listed in Table S4, which can be explained by the removal of active slits. This cell was further tuned to exhibit monostability (ODC_M_) by increasing the slit width (*w_1_*) to 1.1 mm, which was selected based on the response surface shown in Figure 2g. Figure 6b shows the corresponding force‐displacement responses, confirming that ODC_M_ does not reach a secondary stable state, in contrast to other bistable ODC configurations. Furthermore, the lower critical force of ODC_M_ (1.72 N) compared with the other ODC configurations facilitates simultaneous cold programming of ODCM together with the neighboring bistable cells.

Multimodal mechanisms were realized by coupling these cells with distinct stability and material characteristics. A double‐layer bridging structure was designed (Figure 6c), in which each layer consists of four serially connected ODC units joined by flexible connectors. This architecture enables both mechanically and thermally programmed deformation modes. The programming sequence and subsequent deformation behavior of this bridge structure are governed by the combination of bistability and material composition, of which detailed kinetics responses are summarized in Table S4.

Figure 6d presents a mechanically programmed bridging mechanism composed of bistable ODC_3_ (top layer) and monostable ODC_M_ (bottom layer). Upon tensile loading exceeding the threshold force, the ODC_3_ cells undergo bistable expansion and retain their elongated state, whereas the ODC_M_ cells elastically recover upon unloading (d2). This deformation mismatch induces an upward curvature, forming a cold‐programmed arch (d3). Subsequent heating at 70°C triggers recovery of the ODC_3_ cells (d4), restoring the structure to a flat configuration (d5).

In contrast, a thermally programmed inverse‐bridging mechanism is shown in Figure 6e. In this configuration, the upper layer comprises passive ODC_P_ cells, while the lower layer consists of ODC_3_ cells. Following tensile loading as cold programming, both layers remain in bistably expanded states (e3). Upon subsequent heating, only the ODC_3_ layer recovers, while the ODC_P_ layer remains fixed (e4), resulting in upward bending and bridge formation (e5). The contrasting deformation modes of these configurations are compared in Movie S7.

Compressive testing was conducted to evaluate the mechanical functionality of the bistable bridges (Figure 6f). The force‐displacement curve exhibits a peak force of 0.98 N at a displacement of 35 mm, corresponding to arch flattening. Beyond this point, a negative‐stiffness region appears, where the force decreases with increasing displacement. This behavior suggests potential applications in energy absorption and impact‐mitigation, achievable through both mechanical and thermal activation.

These results establish a design rule in which stability mismatch and material‐dependent recovery govern deformation pathways and structural functions. By combining bistable and monostable units with tailored thermomechanical responses, multimodal and reconfigurable mechanisms can be systematically designed for functionally deployable systems.

### 2.7 Multistable 4D Kirigami Grippers with Programmable Inward/outward Grasping

**FIGURE 7:**
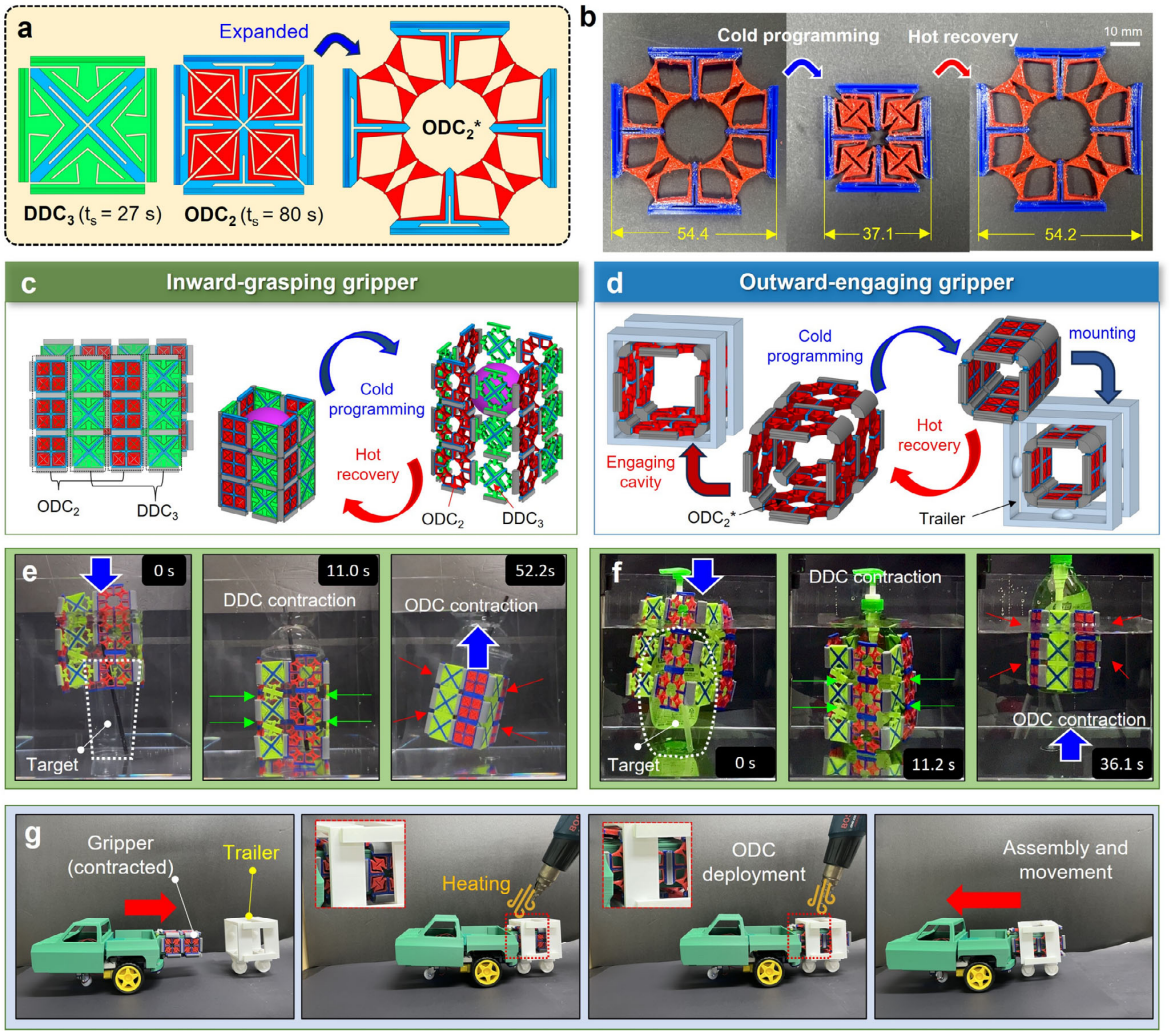
Multistable 4D kirigami grippers with programmable inward and outward grasping. (a) Kirigami unit cells used in gripper design (DDC_3_, ODC_2_, and ODC_2_^*^), where ODC_2_^*^ denotes the exploded configuration of ODC_2_. (b) Cold‐programmed bistable contraction and thermally triggered recovery of ODC_2_^*^, with the corresponding dimensional changes (unit: mm). (c) Inward‐grasping gripper assembled from a 3 × 8 kirigami array, designed to contract from an expanded state upon thermal activation. (d) Outward‐engaging gripper assembled from a 2 × 8 kirigami array printed in the expanded configuration and operated by inverting the transformation pathway. (e) Sequential inward grasping of a tapered cylindrical object enabled by staged DDC and ODC recovery. (f) Shape‐adaptive grasping of a non‐circular object through coordinated multiaxial contraction. (g) Outward grasping (deploy‐to‐engage) mechanism demonstrating mechanical coupling with a confined structure.

Building on the programmable multistable deformation and sequential 4D recovery of composite kirigami assemblies, a box‐type gripper was designed to demonstrate programmable inward and outward grasping. The inward grasping mode exploits two kirigami cells with distinct recovery kinetics, DDC_3_ (*t_s_* = 27.4 s) and ODC_2_ (*t_s_* = 80.0 s), to achieve sequential contraction (Figure 7a). In contrast, the outward grasping mode employs ODC_2_ cells printed in an expanded configuration (denoted ODC_2_^*^), which can be cold‐programmed into a contracted state and subsequently recover to the expanded configuration upon thermal activation (Figure 7b).

For inward grasping, the gripper was constructed from a stripe‐patterned 3 × 8 kirigami array (Figure 7c). Each face comprises a 3×2 arrangement of alternating DDC_3_ and ODC_2_ cells, enclosing an internal cavity of 78 × 78 mm. Following the programming sequence demonstrated in Figure 4d,f, the DDC_3_ cells (*F_crit_* = 1.52 N) first underwent uniaxial snap‐through into the expanded secondary stable state. As the applied displacement increased further, the ODC_2_ cells (*F_crit_* = 4.67 N) subsequently underwent diagonal snap‐through, resulting in the bistably expanded configuration of the entire gripper. After unloading, the programmed gripper retained this expanded configuration (approximately 110 × 110 mm) without external constraints. Upon heating, it recovered to its contracted configuration, thereby functioning as a thermally actuated shrinkable gripper.

Figure 7e illustrates the grasping sequence for a tapered plastic cup (maximum diameter 97 mm; minimum 60 mm). Upon immersion in a 70°C water bath, the DDC layer recovers first, generating horizontal contraction that initiates lateral contact. The delayed recovery of the ODC layer subsequently induces vertical contraction, completing a biaxial enclosure. This coordinated two‐stage contraction enables secure grasping of tapered geometries.

The same mechanism also enables grasping of objects with non‐circular cross‐sections, such as a pump dispenser (114 × 74 mm; Figure 7f). Initial DDC‐driven contraction constrains the longer axis (∼94 × 94 mm), followed by ODC‐driven contraction that completes the enclosure of the irregular geometry. The sequential grasping mechanisms for both objects are shown in Movie S8, demonstrating shape‐adaptable gripping without additional sensors or actively controlled actuators.

For outward grasping, ODC_2_^*^ cells printed in an expanded configuration were cold‐programmed into a contracted state (Figure 7d). To investigate the shape transformation mechanics of ODC_2_^*^, an additional FEA was conducted to analyze its cold‐programming behavior, and the results are presented in Figure S4. In particular, Figure S4b compares the strain‐energy responses of the closed‐as‐printed ODC_2_ and open‐as‐printed ODC_2_^*^ configurations. Although ODC_2_^*^ reaches its second stable state at a similar apparent strain (*ε_a_* = 0.61) to ODC_2_ (*ε_a_* = 0.63), both *F_crit_* and *ΔE* are considerably higher for ODC_2_^*^. These results indicate that the open‐as‐printed configuration requires a larger programming load and forms a more stable bistable state. Moreover, the stepwise deformation sequence (S_1_–S_4_) and the corresponding evolution of strain‐energy density of ODC_2_^*^ are presented in Figure S4c. The results show that ODC_2_^*^ passes through a highly unstable, buckling‐induced configuration before reaching the secondary stable state, which contributes to its higher *F*_crit_ and Δ*E* values compared with ODC_2_.

The assembled gripper was mounted to the rear of a miniature tractor and inserted into a trailer cavity (Figure 7g). Upon heating with 70°C hot air, this structure recovered to its expanded configuration, mechanically engaging the internal wall and locking the connection. This deploy‐to‐engage mechanism enables robust mechanical coupling.

Together, these inward (shrink‐to‐grasp) and outward (deploy‐to‐engage) modes demonstrate versatile manipulation enabled by modular multistable kirigami assemblies. This establishes a design rule in which the recovery kinetics and transformation pathways, programmed at the unit‐cell level, govern macroscopic grasping modes and interaction strategies. Such electronics‐free actuation is particularly attractive for soft robotic systems requiring simplicity, robustness, and low power consumption [40].

### 2.8 Biomimetic Underwater Robot with Autonomous Navigation and Cleaning

**FIGURE 8:**
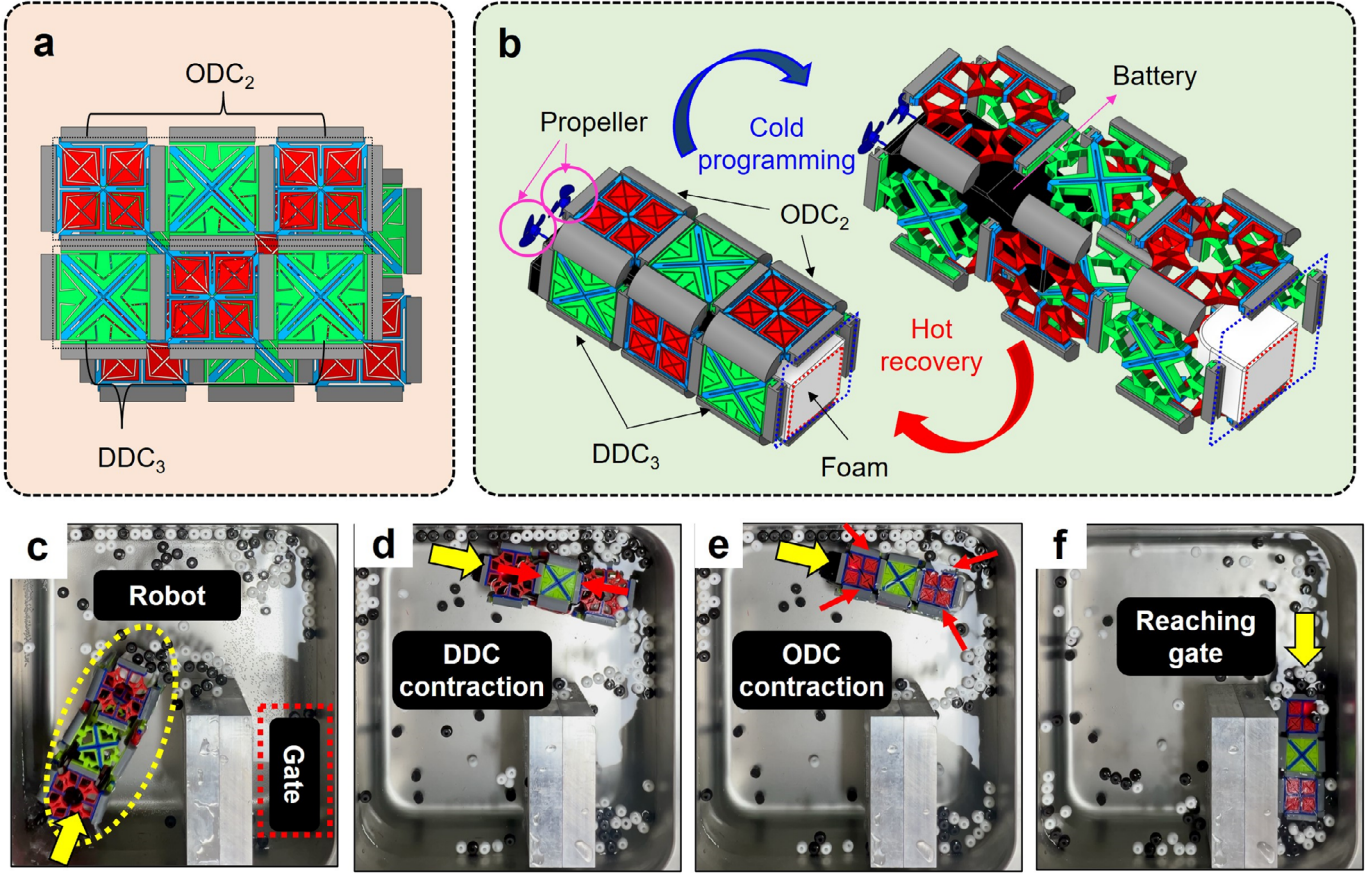
Biomimetic underwater cleaning robot with enabled by sequential 4D recovery of a composite kirigami assembly. (a) Design of an underwater cleaning robot based on a checkerboard‐patterned 4 × 3 kirigami array, integrating DDC_3_ and ODC_2_ cells for programmable sequential recovery. (b) Folding into a 1 × 1 × 3 box configuration (50 × 50 × 120 mm) and cold programming to a bistably expanded state (∼66 × 66 × 172 mm), mounted by a waterproof DC motor with dual side propellers. (c) Expanded swimming and debris captures prior to thermal activation. (d) Thermally triggered first‐stage contraction: DDC‐driven longitudinal collapse initiating closure. (e) Second‐stage contraction: delayed bidirectional recovery of ODC layers completing enclosure of the collected payload. (f) Fully contracted configuration (50 × 50 mm cross‐section) enabling reduced drag and traversal through confined passages (e.g., 71 mm gate) for payload delivery.

The box‐type composite kirigami architecture was further extended to realize a biomimetic underwater robot with autonomous cleaning functions. To program sequential 4D recovery, the robot was constructed from a checkerboard‐patterned 4 × 3 kirigami array (Figure 8a), by integrating DDC_3_ and ODC_2_ cells. The same DDC_3_ and ODC_2_ configurations used in the inward gripper described in Section 2.7 were employed; consequently, the cold‐programming process followed the same sequence, with uniaxial snap‐through of DDC_3_ occurring first, followed by diagonal snap‐through of ODC_2_. The planar array was folded into a 3D box configuration with a 1 × 1 × 3 stacking arrangement (50 × 50 × 120 mm), and the assembled structure was subsequently cold‐programmed into a bistably expanded configuration (∼66 × 66 × 172 mm), which was retained after unloading, as shown in Figure 8b. A rear‐mounted waterproof DC motor with dual side propellers provides active propulsion for underwater navigation.

The operational sequence begins with the robot in its expanded configuration, during which it swims and captures floating debris (e.g., 11.8 mm plastic beads) through the open intake (Figure 8c). Upon thermal activation, the DDC layer contracts first, inducing partial longitudinal contraction (Figure 8d). Subsequently, the delayed bidirectional contraction of the ODC layers completes closure and encloses the collected payload within the robot body (Figure 8e). This stepwise reconfiguration also reduces the robot's cross‐sectional area to 50×50 mm, thereby reducing hydrodynamic drag and enhancing post‐collection swimming stability and efficiency. The resulting compact form enables traversal through narrow passages (e.g., 71 mm gates) to deliver the payload to the designated location (Figure 8f). The full sequence of these operations is provided in Movie S9.

Overall, this underwater cleaning robot leverages the 2D‐to‐3D transformation capabilities of modular kirigami systems to enable a functional robotic platform. The key behaviors—shape programming, debris capture, enclosure, and reconfiguration—are mechanically encoded and triggered thermally, without requiring additional sensors or electronic control. This mechanically programmed strategy supports untethered, low‐power operation suited for long‐duration tasks in unstructured or submerged environments. While the current prototype uses three stacked layers, extending the modular architecture and programming distinct recovery thresholds across segments could enable more complex, snake‐like locomotion and navigation.

## 3 Conclusions

This work established a modular kirigami‐based framework for programmable multistable deformation and sequential 4D actuation driven by mechanical programming and thermal activation. Two kirigami unit cells, inspired by Korean *Dancheong* motifs and realized through active/passive thermoplastic integration, enabled cold‐programmed bistability and sequentially controlled recovery. By tuning geometric parameters and material distribution, both the bistable energy landscape and recovery kinetics can be systematically programmed.

Building on this framework, modular assemblies of these unit cells enabled hierarchical multistability and spatiotemporally programmable actuation. Particularly, the decoupling of mechanically defined bistable states from thermally governed recovery kinetics allowed independent control over deformation pathways and recovery sequences. This principle was directly translated into three functional mechanisms: (i) multimodal bridging structures capable of both mechanically and thermally induced reconfigurations; (ii) multistable 4D grippers capable of inward (shrink‐to‐grasp) and outward (deploy‐to‐engage) transformations; and (iii) a biomimetic underwater cleaning robot that autonomously collects and encloses floating debris through sequential thermal recovery.

Compared with conventional soft actuators relying on embedded electronics or pneumatic system, the proposed approach enables electronics‐free actuation through material‐structure coupling. Furthermore, the use of thermoplastics provides higher stiffness than elastomer‐based actuators, offering improved mechanical robustness while retaining reconfigurability. These features position modular kirigami metamaterials as a promising platform for low‐power, mechanically encoded adaptive mechanisms.

One current limitation of the present MEX‐based fabrication approach is the minimum printable hinge width of 0.4 mm imposed by the nozzle diameter, which constrains geometric miniaturization and deformation resolution. Future work will therefore focus on extending this framework to higher‐resolution multi‐material additive manufacturing processes, such as material jetting [41] or vat photo‐polymerization [42], to achieve finer hinge architectures, enhanced geometric complexity, and more sophisticated actuation behaviors.

While the present study focuses on two representative kirigami architectures, namely DDC and ODC, the results reveal general design principles linking geometry‐controlled multistability and material‐dependent recovery kinetics. Within the proposed DDC–ODC framework, geometry governs bistable deformation, material distribution dictates recovery behavior, and their coupling defines macroscopic deformation modes and programmable functions. By bridging architected kirigami metamaterials and dual‐material 4D printing, this framework provides a scalable manufacturing strategy for thermo‐mechanically encoded actuation in adaptive mechanisms. Future studies may extend these principles to other kirigami architectures and material systems to further broaden the range of achievable mechanical functions.

## 4 Experimental Section

### 4.1 Materials

Two thermoplastic filaments, polylactic acid (PLA) and thermoplastic polyurethane (TPU), were used. PLA (RAL5002, UltiMaker, Netherlands) was selected as the thermally active material, while TPU (Filaflex 82A, Recreus Industries, Spain) served as the passive structural material. At room temperature, PLA exhibits a high elastic modulus of 3250 MPa and a tensile strength of 52.5 MPa while TPU has a substantially lower elastic modulus of 22 MPa and a tensile strength of 45 MPa. These tensile strength values were subsequently used to assess the structural integrity of the kirigami cells under large deformation conditions. Dynamic mechanical analysis (DMA) revealed that the storage modulus of PLA decreases sharply above *T_g_* (64.4°C), falling below that of TPU (Figure S5). This modulus crossover enables selective softening of the active regions, allowing the release of stored strain energy and driving thermal recovery. Based on this thermomechanical contrast, PLA and TPU were combined to construct kirigami unit cells with spatially defined active and passive domains, enabling programmable bistability and thermally triggered actuation.

### 4.2 Additive Manufacturing

All kirigami cells (30 × 30 × 4 mm) were fabricated using a dual‐nozzle material extrusion (MEX)‐type 3D printer (Epsilon W27, BCN3D, Spain). A nozzle diameter of 0.4 mm and a layer thickness of 0.2 mm were used to ensure geometric fidelity. Owing to the nozzle diameter limitation, the minimum hinge width in the kirigami structures was constrained to 0.4 mm to ensure reliable extrusion and structural continuity during printing. PLA was printed at 230°C with a speed of 60 mm/s, while TPU was printed at 240°C and 45 mm/s to accommodate its lower viscosity. To promote interfacial bonding, a 0.3 mm overlap was introduced at the PLA‐TPU interface regions.

### 4.3 Cold Programming and Thermally Triggered 4D Recovery

Cold‐programming was performed at room temperature by mechanically deforming the printed kirigami cells into their second stable configurations until completion of the snap‐through transition. After unloading, the programmed cells retained their bistable configurations without external constraints. Thermal recovery was induced by immersing the programmed structure in a 70°C water bath, exceeding *T_g_* of PLA (64.4°C). The recovery process was recorded in real time until the structures returned to their primary stable states. After recovery, specimens were cooled to room temperature for 5 min prior to subsequent testing or reuse. Recovery time (*t_s_*) was defined as the duration required to reach the initial stable configuration. To evaluate the reliability of bistable‐state retention and thermal recovery under repeated thermomechanical loading, five consecutive cold‐programming–thermal‐recovery cycles were performed on representative DDC_3_ and ODC_2_ unit cells. Four independently fabricated specimens were tested for each architecture. In each cycle, the specimens were cold‐programmed into the second stable state and unloaded to verify bistable‐state retention. The recovered length (*l*_r_), residual strain (*ε_r_*), and recovery time (*t*_s_) were measured after each recovery cycle.

### 4.4 Finite Element Analysis

FEAs were conducted using ANSYS Mechanical (ANSYS Inc., USA) to investigate the bistable deformation of kirigami unit cells. A 2D plane‐stress model with eight‐node quadrilateral elements was employed to accurately capture deformation in the thin hinge region. Active and passive domains were explicitly defined through geometric parameters, including the active slit length (*l_a_*), slit width (*w_1_*), and hinge width (*w_h_*), to evaluate their influence on bistability. Uniaxial tensile loading was simulated by applying incremental horizontal displacement until snap‐through transitions occurred, followed by an additional extension of 1.0 mm. The relevant boundary conditions and mesh details are provided in Figure S6.

### 4.5 Tensile Tests for Kirigami Assemblies

Uniaxial tensile tests were conducted to evaluate the multistable behavior of composite kirigami assemblies composed of homogeneous and heterogeneous arrangements of DDC and ODC units (Figure S7). Prior to testing, individual kirigami cells were assembled using dovetail‐shaped boundaries, and modular connectors were inserted between adjacent cells to ensure mechanical interlocking. The connectors were fabricated from polymethyl methacrylate (PMMA, *T_g_* = 105°C) to maintain structural integrity during thermal activation and recovery. Mechanical testing was performed using a universal testing machine (NA‐2M, Nanotech, Korea) at a crosshead speed of 10 mm/min. The top and bottom edges were clamped, while lateral sliding was permitted to accommodate auxetic deformation. Force–displacement data and deformation sequences were recorded to characterize collective multistability and sequential snap‐through behavior.

## Author Contributions

**Eui‐Hyun Kim**: conceptualization, methodology, investigation, visualization, software, validation, data curation, Writing – original draft. **Keun Park**: conceptualization, methodology, funding acquisition, project administration, supervision, writing – original draft, writing – review and editing. **Hyunsik Yoon**: conceptualization, visualization, writing – review and editing, formal analysis. **Minsung Kim**: investigation, visualization, validation, resources.

## Funding

This research was financially supported by the National Research Foundation of Korea (NRF) grant funded by the Ministry of Science and ICT, Republic of Korea (Grant numbers: RS‐2024‐00333963; RS‐2025‐00512549).

## Conflicts of Interest

The authors declare no conflicts of interest.

## Declaration of the Use of AI

The authors declare that artificial intelligence–based tools were used in a limited manner during the preparation of this manuscript. Specifically, ChatGPT 5.0 was utilized to assist with language editing and clarity improvement of the text. All scientific content, including the problem formulation, methodology, data analysis, results, and conclusions, was conceived, verified, and validated by the authors. The authors take full responsibility for the integrity, originality, and accuracy of the work.

## Supporting information




**Supporting File1**: advs77412‐sup‐0001‐SuppMat.docx.

**Supporting File2**: advs77412‐sup‐0002‐MovieS1.mp4.

**Supporting File3**: advs77412‐sup‐0003‐MovieS2.mp4.

**Supporting File1**: advs77412‐sup‐0004‐MovieS3.mp4.

**Supporting File1**: advs77412‐sup‐0005‐MovieS4.mp4.

**Supporting File2**: advs77412‐sup‐0006‐MovieS5.mp4.

**Supporting File3**: advs77412‐sup‐0007‐MovieS6.mp4.

**Supporting File4**: advs77412‐sup‐0008‐MovieS7.mp4.

**Supporting File5**: advs77412‐sup‐0009‐MovieS8.mp4.

**Supporting File6**: advs77412‐sup‐00010‐MovieS9.mp4.

## Data Availability

The data that supports the findings of this study are available in the supplementary material of this article.
