# Supplementary material for: Programmable Sequential 4D Actuation in Multistable Kirigami Metamaterials
Source: Adv Sci (Weinh). 2026 Aug 24:e77412. Online ahead of print. doi: 10.1002/advs.77412 (PMC13502354; doi:10.1002/advs.77412)
Supplement: Supplementary file 1 [file ADVS-9999-e77412-s004.docx]

Supplementary Information

Programmable Sequential 4D Actuation in Multistable Kirigami Metamaterials

Eui-Hyun Kim, Minsung Kim, Hyunsik Yoon, and Keun Park^*^

1. **Supplementary Figures** (Figures S1–S7)

- **Figure S1.** Detailed design parameters of kirigami unit cells: a) DDC and b) ODC.
- **Figure S2.** Maximum stress variations at selected hinge points for: a) the DDC and b) the ODC. Detailed effective stress distributions at key bistable states (S_1_–S_4_) for: c) the DDC and d) the ODC.
- **Figure S3.** Effects of hinge width (*w_h_*) on the bistability behavior for DDC: a) Force response and b) Strain-energy response. Effects of slit width (*w_s_*) on the bistability behavior for ODC: c) Force response and d) Strain-energy response.
- **Figure S4**. FEA results of the cold-programming behavior of open-as-printed ODC_2_^*^ configurations. a) Definition of analysis domain and boundary conditions (bidirectional compression). b) Strain-energy responses of the closed-as-printed ODC_2_ and open-as-printed ODC_2_^*^ configurations. c) Stepwise deformation sequence (S_1_–S_4_) and the corresponding evolution of strain-energy density of ODC_2_^*^.
- **Figure S5.** The dynamic mechanical analysis (DMA) test results for PLA and TPU. a) storage modulus (*E’*). b) loss factor (tan *δ*).
- **Figure S6.** Finite element analysis models and boundary conditions for kirigami cells: a) DDC (3,083 nodes and 2,758 elements). b) ODC (6,106 nodes and 5,544 elements).
- **Figure S7.** Multistable deformation of homogeneous and heterogeneous array structures. a) Assembly of unit cells using dovetail connectors. b) Homogeneous cell arrays of DDC and ODC. c) Heterogeneous checkerboard and stripe composites alternating DDC and ODC units. d) Tensile test setup for the stripe-type kirigami assembly, wherein lateral sliding was allowed to facilitate auxetic expansion.

2. **Supplementary Tables** (Tables S1–S4)

- **Table S1.** Design parameters of the DDC with their ranges and reference values.
- **Table S2.** Design parameters of the ODC with their ranges and reference values.
- **Table S3.** Geometric parameters and thermo-mechanical responses of various DDC designs.
- **Table S4.** Geometric parameters and thermo-mechanical responses of various ODC designs.
- **Table S5.** Recovered length (*l*_r_), residual strain (*ε_r_*), and recovery time (*t*_s_) for DDC_3_ and ODC_2_ unit cells under repeated thermomechanical cycles.

3. **Supplementary Movies** (Movies S1–S9)

- **Movie S1.** Bistable deformation sequence of a homogeneous DDC array.
- **Movie S2.** Bistable deformation sequence of a homogeneous ODC array.
- **Movie S3.** Multistable deformation sequence of heterogeneous composite structures (checkerboard configurations).
- **Movie S4.** Multistable deformation sequence of heterogeneous composite structures (stripe configurations).
- **Movie S5.** Thermally triggered 4D recovery of checkerboard composite structures (DDC-first recovery).
- **Movie S6.** Thermally triggered 4D recovery of checkerboard composite structures (ODC-first recovery).
- **Movie S7.** Cold programming and 4D recovery behavior for mechanically and thermally actuating bridge structures.
- **Movie S8.** Sequential contraction mechanisms of the box-type gripper for grasping various objects.
- **Movie S9.** Operation sequence of the underwater cleaning robot, including swimming, capturing debris, enclosing, and delivery.

**Figure S1.** Detailed design parameters of kirigami unit cells (unit: mm): a) DDC and b) ODC, wherein the parameters for each cell and their ranges are defined in Tables S1 and S2, respectively.

**Figure S2.** Maximum stress variations at selected hinge points (P_0_–P_2_) for: a) the DDC and b) the ODC. Detailed effective stress distributions at key bistable states (S_1_–S_4_) for: c) the DDC and d) the ODC. Overall, the ODC exhibits higher stress levels than the DDC, with stress concentration distributed across multiple hinge locations. In the DDC, the passive-region hinges (P_1_ and P_2_) reach their peak stresses at state S_2_, whereas in the ODC, peak stresses at passive hinges occur between S_2_ and S_3_. In contrast, the active hinge (P_0_) exhibits the peak stress between S_1_ and S_2_ in both cell types, with magnitudes consistently higher than those at the passive hinges.

**Figure S3.** Effects of hinge width (*w_h_*) on the bistability behavior for DDC: a) Force response and b) Strain-energy response. The *w_h_* = 1.2 mm case shows the monostable response while the other two cases show bistable responses. Effects of slit width (*w_s_*) on the bistability behavior for ODC: c) Force response and d) Strain-energy response. The *w_h_* = 1.1 mm case shows the monostable response while the other two cases show bistable responses. These results highlight that the kirigami design parameters should be tuned to ensure bitstability.

**Figure S4.** FEA results of the cold-programming behavior of open-as-printed ODC_2_^*^ configurations. a) Definition of analysis domain and boundary conditions (bidirectional compression). b) Strain-energy responses of the closed-as-printed ODC_2_ and open-as-printed ODC_2_^*^ configurations, with energy barriers of 8.02 and 20.03 N/mm², respectively. c) Stepwise deformation sequence (S_1_–S_4_) and the corresponding evolution of strain-energy density of ODC_2_^*^, with enlarged views of hinges P_1_ and P_2_ that represent a sequential buckling (S_2_) and release (S_3_).

**Figure S5.** The dynamic mechanical analysis (DMA) test results for PLA and TPU. a) storage modulus (*E’*). b) loss factor (tan *δ*). While the storage modulus of TPU is 15.5 MPa at room temperature and decreases to 5.27 MPa at 83°C, PLA shows a significant change in storage modulus, from 2292 MPa at room temperature to 4.02 MPa at 83°C, and the glass transition temperature (*T_g_*) of 64.4°C. Based on these results, PLA was selected as an active thermoresponsible material while TPU was used as a passive material.

**Figure S6.** Finite element analysis models and boundary conditions for kirigami cells. a) the DDC model consisting of 3,083 nodes and 2,758 elements. b) the ODC model consisting of 6,106 nodes and 5,544 elements. A quarter domain for each cell was selected and discretized considering symmetry. The global mesh size was set to 0.5 mm, whereas the cell hinge regions were refined to 0.1 mm. A horizontal displacement was applied until the deformation reached the bistable state, with an additional 1.0 mm displacement afterward.

**Figure S7.** Multistable deformation of homogeneous and heterogeneous array structures. a) Assembly of unit cells using dovetail connectors. b) Homogeneous cell arrays of DDC and ODC. c) Heterogeneous checkerboard and stripe composites alternating DDC and ODC units. d) Tensile test setup for the stripe-type kirigami assembly, wherein lateral sliding was allowed to facilitate auxetic expansion.

**Table S1.** Design parameters of the DDC with their ranges and reference values.

| Parameter | Description (unit) | Range | Reference |
| --- | --- | --- | --- |
| *l_a_* | Active slit length (mm) | 10 – 16 | 16 |
| *w_h_* | Hinge width (mm) | 0.4 – 0.8 | 0.4 |
| *t_1_* | Leg-1 thickness (mm) | 0.8 – 8.0 | 1.6 |
| *t_2_* | Leg-1 thickness (mm) | 0.8 – 5.6 | 2.0 |
| *θ_1_* | Slit-1 angle (degree) | 20 – 60 | 40 |
| *θ_2_* | Slit-2 angle (degree) | -20 – 60 | 5.0 |

**Table S2.** Design parameters of the ODC with their ranges and reference values.

| Parameter | Description (unit) | Range | Reference |
| --- | --- | --- | --- |
| *l_a_* | Active slit length (mm) | 6 – 12 | 10 |
| *d_s_* | Slit position (mm) | 0.4 – 2.8 | 0.4 |
| *w_1_* | Slit-1 width (mm) | 0.3 – 0.7 | 0.5 |
| *w_2_* | Slit-2 width (mm) | 0.4 – 1.2 | 0.6 |
| *θ* | Slit angle (degree) | -5 – 15 | 5.0 |

**Table S3.** Geometric parameters and thermo-mechanical responses of various DDC designs.

| Parameter | DDC_1_ | DDC_2_ | DDC_3_ | DDC_4_ |
| --- | --- | --- | --- | --- |
| *l_a_* (mm) | 10 | 12 | 14 | 16 |
| *w_h_* (mm) | 0.4 | 0.4 | 0.4 | 0.4 |
| *t_1_* (mm) | 1.6 | 1.6 | 1.6 | 1.6 |
| *t_2_* (mm) | 2.0 | 2.0 | 2.0 | 2.0 |
| *θ_1_* (degree) | 40 | 40 | 40 | 40 |
| *θ_2_* (degree) | 5.0 | 5.0 | 5.0 | 5.0 |
| *F_crit_* (N) | 1.57 | 1.56 | 1.52 | 1.49 |
| *ΔE* (N/mm^2^) | 1.99 | 1.96 | 1.93 | 1.88 |
| *t_s_* (second) | N/A | 52.0±10.1 | 27.4±1.4 | 22.0±1.7 |

**Table S4.** Geometric parameters and thermo-mechanical responses of various ODC designs.

| Cell type | ODC_1_ | ODC_2_ | ODC_3_ | ODC_4_ | ODC_P_ | ODC_M_ |
| --- | --- | --- | --- | --- | --- | --- |
| *l_a_* (mm) | 6 | 8 | 10 | 12 | 0 | 0 |
| *d_s_* (mm) | 0.4 | 0.4 | 0.4 | 0.4 | 0.4 | 0.4 |
| *w_1_* (mm) | 0.5 | 0.5 | 0.5 | 0.5 | 0.5 | 1.1 |
| *w_2_* (mm) | 0.6 | 0.6 | 0.6 | 0.6 | 0.6 | 0.6 |
| *θ* (degree) | 5.0 | 5.0 | 5.0 | 5.0 | 5.0 | 5.0 |
| *F_crit_* (N) | 4.86 | 4.67 | 4.46 | 4.10 | 4.95 | 1.72 |
| *ΔE* (N/mm^2^) | 8.71 | 8.47 | 8.02 | 7.34 | 8.82 | N/A |
| *t_s_* (second) | N/A | 80.0±3.6 | 24.3±1.5 | 12.3±0.6 | N/A | N/A |

**Table S5.** Recovered length (*l*_r_), residual strain (*ε_r_*), and recovery time (*t*_s_) for DDC_3_ and ODC_2_ unit cells under repeated thermomechanical cycles.

| Cycle | DDC_3_ | | | ODC_2_ | | |
| --- | --- | --- | --- | --- | --- | --- |
|  | $l_{r}$(mm) | $\varepsilon_{r}$(%) | $t_{s}$ (s) | $l_{r}$(mm) | $\varepsilon_{r}$(%) | $t_{s}$ (s) |
| 1 | 30.5±0.1 | 1.54±0.48 | 27.4±1.4 | 31.9±0.2 | 6.33±0.73 | 80.0±3.6 |
| 2 | 30.6±0.2 | 1.83±0.58 | 26.1±3.0 | 32.3±0.1 | 7.77±0.34 | 68.4±7.3 |
| 3 | 30.5±0.1 | 1.77±0.28 | 25.4±2.4 | 32.6±0.3 | 8.66±0.98 | 50.5±2.7 |
| 4 | 30.6±0.1 | 2.12±0.45 | 26.7±3.9 | 32.5±0.2 | 8.32±0.60 | 46.6±7.0 |
| 5 | 30.8±0.0 | 2.65±0.09 | 25.0±3.8 | 32.5±0.3 | 8.43±0.99 | 41.8±2.0 |
